# VEGF-A165 is the predominant VEGF-A isoform in platelets, while VEGF-A121 is abundant in serum and plasma from healthy individuals

**DOI:** 10.1371/journal.pone.0284131

**Published:** 2023-04-07

**Authors:** Munekazu Yamakuchi, Masashi Okawa, Kazunori Takenouchi, Aryal Bibek, Shingo Yamada, Keiichi Inoue, Kazuhiko Higurashi, Akito Tabaru, Kiyonori Tanoue, Yoko Oyama, Sadayuki Higashi, Chieko Fujisaki, Hideaki Kanda, Hiroto Terasaki, Taiji Sakamoto, Yoshiharu Soga, Teruto Hashiguchi

**Affiliations:** 1 Department of Laboratory and Vascular Medicine, Graduate School of Medical and Dental Sciences, Kagoshima University, Kagoshima, Japan; 2 Department of Cardiovascular Surgery, Graduate School of Medical and Dental Sciences, Kagoshima University, Kagoshima, Japan; 3 Shino-Test Corporation, Sagamihara, Japan; 4 Kagoshima University Hospital Clinical Laboratory, Kagoshima, Japan; 5 Department of Ophthalmology, Kagoshima University Graduate School of Medical and Dental Sciences, Kagoshima, Japan; TotiCell Limited, Bangladesh, BANGLADESH

## Abstract

Vascular endothelial growth factor A (VEGF-A) plays pivotal roles in regulating tumor angiogenesis as well as physiological vascular function. The major VEGF-A isoforms, VEGF-A121 and VEGF-A165, in serum, plasma, and platelets have not been exactly evaluated due to the lack of the appropriate assay system. Antibodies against human VEGF-A121 and VEGF-A165 (hVEGF-A121 and hVEGF-A165) were successfully produced and Enzyme-Linked ImmunoSorbent Assay (ELISA) for hVEGF-A121 and hVEGF-A165 were separately created by these monoclonal antibodies. The measurement of recombinant hVEGF-A121 and hVEGF-A165 by the created ELISA showed no cross-reaction between hVEGF-A121 and hVEGF-A165 in conditioned media from HEK293 cells transfected with either hVEGF-A121 or hVEGF-A165 expression vector. The levels of VEGF-A121 and VEGF-A165 in serum, plasma, and platelets from 59 healthy volunteers proved that VEGF-A121 level was higher than VEGF-A165 in both plasma and serum in all the cases. VEGF-A121 or VEGF-A165 in serum represented higher level than that in plasma. In contrast, the level of VEGF-A165 was higher than VEGF-A121 in platelets. The newly developed ELISAs for hVEGF-A121 and hVEGF-A165 revealed different ratios of VEGF isoforms in serum, plasma, and platelets. Measuring these isoforms in combination provides useful information as biomarkers for diseases involving VEGF-A121 and VEGF-A165.

## Introduction

Vascular endothelial growth factor (VEGF) was discovered as an important growth factor in vasculogenesis and angiogenesis about 30 years ago [1]. At least, five types of human VEGFs, VEGF-A, -B, -C, -D, and placental growth factor (PlGF), have been identified [2, 3]. Each type of VEGF family differentially regulates angiogenesis and lymphangiogenesis by binding to VEGF receptors (VEGFR1-3) and co-receptors (Neuropilins (NRP1-2)) [4, 5].

VEGF-A is the major pro-angiogenic type among these family members [6]. VEGF-A messenger RNA (mRNA) are synthesized from VEGF-A gene, located on chromosome 6p21.3, by alternative exon splicing, leading to produce different isoforms, such as VEGF-A121, VEGF-A165, VEGF-A189, and VEGF-A206 [7]. VEGF-A189 and VEGF-A206 are extracellular matrix or cell membrane-bound types by their two heparin-binding domains, in turn, VEGF-A121 has no heparin-binding domain, which enables VEGF-A121 easily to detect in blood [8]. VEGF-A165 represents in between characteristic because of its single heparin-binding domain and exits in blood. VEGF-A isoforms provide different cellular phenotypes through a variation of VEGFR2—dependent signal transduction, causing to VEGF-A isoform—specific unique biological functions [9]. However, the difference of biological function between VEGF-A121 and VEGF-A165 has not completely been elucidated.

While the utility of evaluating VEGF-A has been recognized in a variety of diseases [10–13], the clinical importance of each VEGF-A isoform has not been confirmed due to the lack of evidence regarding the biological differences between VEGF-A isoforms described above. Another reason is that assay systems for accurately measuring human VEGF-A isoforms are immature. Therefore, it is important to develop a VEGF-A isoform-specific assay system that distinguishes between VEGF-A121 and VEGF-A165.

Platelets play pivotal roles in a variety of pathological processes, such as tumor progression and atherosclerosis as well as physiological hemostatic conditions [14, 15]. VEGF-A in platelets has been shown as potential predictors of the progression of hepatocellular carcinoma [16] or the prognosis in Kawasaki syndrome [17], however, the evaluation of VEGF-A level in platelets, especially VEGF-A121 and VEGF-A165, in healthy individuals has not been addressed yet.

Here we have created a new ELISA system to detect human VEGF-A121 and VEGF-A165 (hVEGF-A121 and hVEGF-A165) separately. The data from these ELISAs showed no cross-reaction between hVEGF-A121 and hVEGF-A165 by a cell-based study. The measurements of VEGF-A121 and VEGF-A165 in plasma, serum, and platelets from healthy volunteers proved the feasibility of the clinical use. In this study, we elucidated that VEGF-165 level was higher than VEGF-A121 in platelets in healthy volunteers, although VEGF-A121 was higher in serum and plasma.

## Materials and methods

### Study participants

Serum and plasma samples were obtained from 59 heathy volunteers who met the criteria of healthy individuals [18]. The research ethics committees of Kagoshima University Hospital approved this study (Approval number 180198). All volunteers provided their written informed consent before being included in the study. The study was conducted in accordance with the ethical standards of the Committee on Human Experimentation of the institution at which the experiments were performed or in accordance with the ethical standards of the Helsinki Declaration of 1975. Venous blood sampling was performed according to the Japanese Committee For Clinical Laboratory Standards (JCCLS) guideline [19].

### Serum and plasma preparation

Whole blood was collected in a serum separating tube (Venoject II, Terumo Corp., Tokyo, Japan), a citrate tube containing 3.2% sodium citrate (Venoject II, Terumo Corp.) for plasma collection, and an EDTA-2K tube (Venoject II, Terumo Corp.) to count cells. The serum separating tube was incubated undisturbed at room temperature for 30 minutes to allow clotting. Both serum and citrate tubes were centrifuged at 1,710 × g for 10 minutes, following at 2,330 × g for another 5 minutes. The supernatants were carefully aliquoted and stored in -80°C.

### Platelet isolation and lysate preparation

Citrate tubes were centrifuged at 90 × g for 15 minutes and gently collected only the top 75% of resultant platelet rich plasma (PRP). Prostaglandin E1 (Cayman Chemical, Ann Arbor, MI) was added (final concentration: 1μM) to inhibit platelet aggregation, and the PRP was recentrifuged at 2,330 × g, and the supernatant was completely removed to separate platelet pellets. Platelet pellets were resuspended in phosphate buffered saline (PBS) with 1μM of prostaglandin E1 to equal volume of PRP and gently washed by pipetting. Washed platelets were recentrifuged at 2,330 × g, and the supernatant was completely removed to reseparate platelet pellets. Platelet pellets were lysed in lysis buffer (Cell Signaling Technology, Danvers, MA) with protease inhibitors (Halt Protease Inhibitor Cocktail EDTA-free; Thermo Fisher Scientific, Waltham, MA) to equal the volume of initially adjusted PRP and immediately stored at -80°C.

### Platelet releasing VEGF-A

Platelet poor plasma (PPP) was prepared from citrate tubes by centrifugation twice at 1,710 × g for 10 minutes and 2,330 × g for 5 minutes. PRP and PPP were stimulated by adding human thrombin (Sigma Chemical Co., St. Louis, MO)/calcium chloride solution (a final concentration: 5 U/ml/10 mM) (PRP+th/Ca^2+^ and PPP+th/Ca^2+^, respectively). After incubation on room temperature for 30 min, these samples were centrifuged at 750 × g for 20 min and the supernatants were measured by ELISA. VEGF-A released from 1 × 10^6^ platelets (Platelet Releasing VEGF-A: PR-VEGF-A) was calculated from the following equation (pg/10^6^ platelets): [(VEGF-A released from PRP: PRP+th/Ca^2+^)–(VEGF-A released from PPP: PPP+th/Ca^2+^)] divided by [(platelet number in PRP)–(platelet number in PPP)].

### Recombinant proteins and plasmids

Recombinant human VEGF-A121 (rhVEGF-A121) and VEGF-A165 (rhVEGF-A165) were purchased from Cell signaling Technologies (Danvers, MA). Recombinant human VEGF-A189 (rhVEGF-A189) and recombinant human VEGF-A206 (rhVEGF-A206) were obtained from ReliaTech GmbH (Wolfenbuttel, Germany) and R&D Biosystems (Minneapolis, MN), respectively. Expression plasmids of hVEGF-A121 and hVEGF-A165 were purchased from Sino Biological (#HG10008-NF, Beijing, China) and OriGene Technologies (#RC229662, Rockville, MD), respectively.

### Preparation of the peptide conjugate for immunization

To create monoclonal antibody to hVEGF-A121 or hVEGF-A165, the specific peptides for each human VEGF-A isoform were selected; ARQEKCDKP (hVEGF-A121) and ARQENP (hVEGF-A165) (S1A Table). This hVEGF-A121 peptide covers the site of exon 5 and exon 8, in turn, hVEGF-A165 peptide consists of exon 5 and exon 7, suggesting that these two peptides exhibit completely their own specific antigen. The peptides corresponding to amino acids (ARQEKCDKP) of hVEGF-A121 and amino acids (ARQENP) of hVEGF-A165 were synthesized by a peptide synthesizer: model 430A (Applied Biosystems, Foster City, CA) by the t-Butoxycarbonylamino acid solid-phase method. Terminal cysteine residues were added after synthesis to allow directional conjugation to a carrier protein, and the peptides were purified and separated by High Performance Liquid Chromatography (HPLC). Keyhole limpet hemocyanin (KLH) (Calbiochem, La Jolla, CA) and bovine serum albumin (BSA) (Sigma) were activated for conjugation with a 10-fold molar excess of m-Maleimidobenzoyl-N-hydroxysuccinimide Ester (Thermo Fisher Scientific). The synthesized peptide was added to m-Maleimidobenzoyl-N-hydroxysuccinimide Ester-BSA (Sigma) and conjugated to activated carriers with a 2-fold molar excess of peptide by incubation at room temperature for 150 minutes. After the reaction was completed, the mixture was dialyzed three times against water, and then freeze-dried.

### Production of monoclonal antibodies of hVEGF-A121 or hVEGF-A165

BALB/c mice were injected initially with 100 μg of the BSA-peptides in Freund’s complete adjuvant intraperitoneally and with 100 μg of hVEGF-A121 peptide (ARQEKCDKP) or hVEGF-A165 peptide (ARQENP) in Freund’s incomplete adjuvant intraperitoneally after 2 weeks. The mice were subsequently boosted with hVEGF-A121 peptide or hVEGF-A165 peptide in saline at 2-week intervals for 2 months and then given a final intravenous injection of saline with hVEGF-A121 peptide or hVEGF-A165 peptide 3 days prior to fusion of immortal myeloma cells with B-cells which produce the appropriate antibody. After fusion was carried out according to the method of Milstein [8], cells from mice peritoneal cavity were resuspended in S clone medium (Sanwa Chemical, Ishikawa, Japan) supplemented with thymidine, aminopterin, and hypoxanthine, and then distributed to the wells of 96-well microtiter plates (Thermo Fisher Scientific). Approximately 2 weeks later, when colonies were visible, the medium was evaluated by ELISA in wells of microtiter plates coated with rhVEGF-A121 and rhVEGF-A165. The cells selected were subcloned until their monoclonality was demonstrated. Antibodies are purified by affinity purification method using Protein A. This study for mice was conducted in strict accordance with the recommendations of the National Institutes of Health’s Guide for the Care and Use of Laboratory Animals. The protocol was managed by Sino-Test Corporation, Kanagawa, Japan. All mice were supplied by Charles River Laboratories Japan (Yokohama, Japan), and mice were housed in a standard housing condition for laboratory animals and provided with food and water ad libitum. All surgical procedure was carried out under sodium pentobarbital anesthesia, and all efforts were made to minimize suffering.

### Cell culture and transfection

HEK293 cells were purchased from American Type Culture Collection (ATCC, Manassas, VA) and cultured in DMEM high glucose media (Gibco, Life Technology, Grand Island, NY) supplemented with 10% fetal bovine serum (FBS) at 37°C and 5% CO_2_. Cells were transfected with hVEGF-A121 or hVEGF-A165 expression plasmid using Lipofectamine 3000 (Invitrogen, Life Technologies, Carlsbad, CA) according to the manufacturer’s instructions. Transfected cells were cultured for the following two days, and the conditioned media were collected.

### Enzyme-linked immunosorbent Assay (ELISA)

Polystyrene microtiter plates were coated and incubated with 100 μL of anti-human VEGF-A polyclonal antibody (#AB-293-NA, R&D Biosystems, Minneapolis, MN) in PBS overnight at 4°C. The plates were washed three times with PBS containing 0.05% Tween 20, and the remaining binding sites in the wells were blocked by incubating the plates for 2 hours with 400 μL/well PBS containing 1% BSA. After the plates were washed, 100 μL of each dilution of the calibrator and samples (1:1 dilution in 0.2 mol/L Tris pH 8.5 and 0.15 mol/L sodium chloride containing 1% Casein) was added to the wells. The plates were then incubated for 15 hours at 25°C. The plates were washed again, and were incubated with 100 μL/well of peroxidase-conjugated anti-human VEGF-A121 or VEGF-A165 peptide monoclonal antibody for 2 hours at 25°C. After another washing step, chromogenic substrate 3,3’,5,5’-tetra-methylbenzidine (Dojindo Laboratories, Kumamoto, Japan) was added to each well. The reaction was terminated with sodium sulfate, and the absorbance at 450 nm was read using a microplate reader (Model 680, Bio-Rad, Irvine, CA). Standard curve was drawn by rhVEGF-A121 or rhVEGF-A165, which were measured linearly in the range of 10–2,000 pg/mL with the chromogenic substrate. Total VEGF-A level was measured by commercial-based ELISA (Human VEGF Quantikine ELISA kit, R&D Biosystems). To compare serum VEGF-A121 concentrations with the current VEGF-A121 ELISA, we purchased another commercial kit for VEGF-A121: VEGF121 ELISA kit (SEB851Hu, Cloud-Clone Corp, Huston, TX).

### Western blotting

Mixtures of each 50 ng of rhVEGF-A121, rhVEGF-A165 and rhVEGF-A189 were loaded on 15% polyacrylamide gels (Bio-Rad) and performed SDS Polyacrylamide Gel Electrophoresis (SDS-PAGE). The gel was transferred to a nitrocellulose membrane (Bio-Rad) at 60 mA for 3 h and the membrane was blocked in PBS containing 1% BSA (Sigma), incubated with 50 ng of monoclonal antibodies against hVEGF-A121 or hVEGF-A165, and VEGF-A polyclonal antibody. After washing with PBS containing 0.05% Tween 20, the membrane was soaked with peroxidase-labeled anti-mouse IgG antibody (Agilent, Santa Clara, CA) in PBS containing 3% BSA. After washing again, the detection of chemiluminescence-labeled bands was performed by ECL western blotting detection reagents (Merck KGaA, Darmstadt, Germany) and the MultiImage II (ProteinSimple, Tokyo, Japan).

### Statistical analysis

Statistical analysis was performed using GraphPad Prism 8 (GraphPad Prism Software, Inc., San Diego, CA). A p-value of <0.05 was considered significant. Spearman’s rank correlation coefficients were used to identify correlations between total VEGF-A and VEGF-A121 or VEGF-A165. Comparisons of VEGF-A121 and VEGF-A165 in serum, plasma, and platelet lysate (PL) and comparison between PR-VEGF-A and VEGF-A in PL (PL-VEGF-A) were analyzed using Wilcoxon rank-sum test.

## Results

### Characterization of monoclonal antibodies against hVEGF-A121 or hVEGF-A165

**Fig 1:**
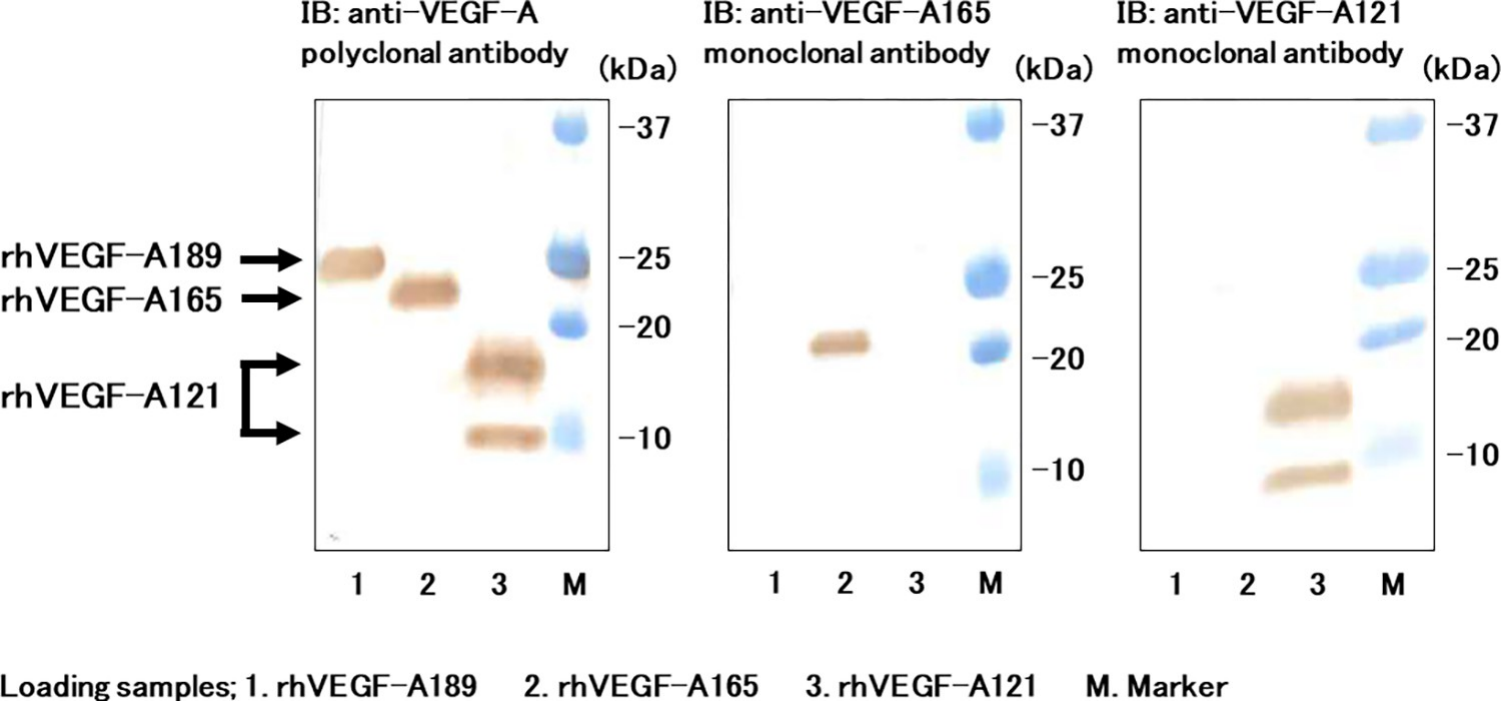
Immunoreactivity of the anti-VEGF-A121 and anti-VEGF-A165 monoclonal antibodies. Mixture of rhVEGF-A121, rhVEGF-A165 and rhVEGF-A189 (50 μg each) were subjected to 15% SDS Polyacrylamide Gel Electrophoresis (SDS-PAGE). The transferred membrane was detected with anti-VEGF-A polyclonal antibody (left panel), anti-VEGF-A165 monoclonal antibody (middle panel), and anti-VEGF-A121 monoclonal antibody (right panel). The upper band of VEGF-A121 bands in SDS-PAGE (Lane 3) is due to glycosylation according to manufacturer’s instruction provided from Cell Signaling Technologies.

At first, the specificity of the anti-human VEGF-A121 or VEGF-A165 monoclonal antibody was examined. Anti-human VEGF-A polyclonal antibody can equally detect rhVEGF-A121, rhVEGF-A165, and rhVEGF-A189 (Fig 1, left panel). The monoclonal VEGF-A121 antibody was capable to react with rhVEGF-A121, but not with rhVEGF-A165 and rhVEGF-A189 by western blotting (Fig 1, right panel). In turn, rhVEGF-A165, but not rhVEGF-A121 and rhVEGF-A189, was detected by the monoclonal VEGF-A165 antibody (Fig 1, middle panel). These data indicated that monoclonal antibody to VEGF-A121 and VEGF-A165 was specific for hVEGF-A121 and hVEGF-A165, respectively. The structures of monoclonal VEGF-A121 and VEGF-A165 antibodies were analyzed by the illumina HiSeq X (Illumina, San Diego, CA). The amino acid sequence of each variable regions in heavy chain and light chain of immunoglobulin G (IgG) were shown in S1B Table.

### Establishment of ELISA for hVEGF-A121 or hVEGF-A165

**Fig 2:**
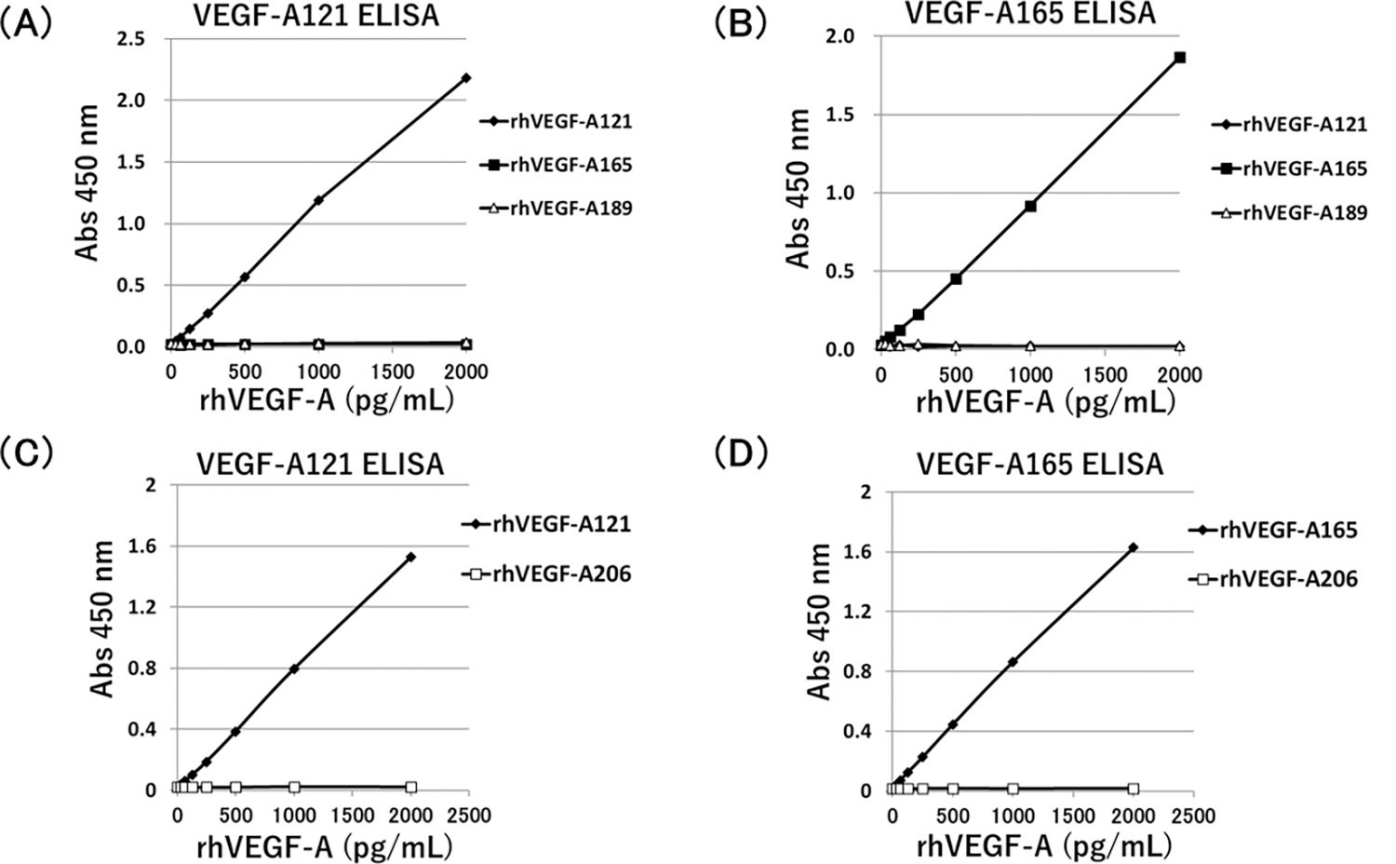
**Specific immunoreactivity of the anti-VEGF-A121 and anti-VEGF-A165 monoclonal antibodies using for ELISA.** The absorbance values at 450 nm (Abs 450 nm) obtained by VEGF-A121 ELISA (A, C) and VEGF-A165 ELISA (B, D). For these ELISAs, different doses of rhVEGF-A121, rhVEGF-A165, and rhVEGF-A189 (A, B) or rhVEGF-A206 (C, D) were evaluated. VEGF-A121 ELISA and VEGF-A165 ELISA specifically recognized rhVEGF-A121 (A, C) and rhVEGF-A165 (B, D), respectively.

**Fig 3:**
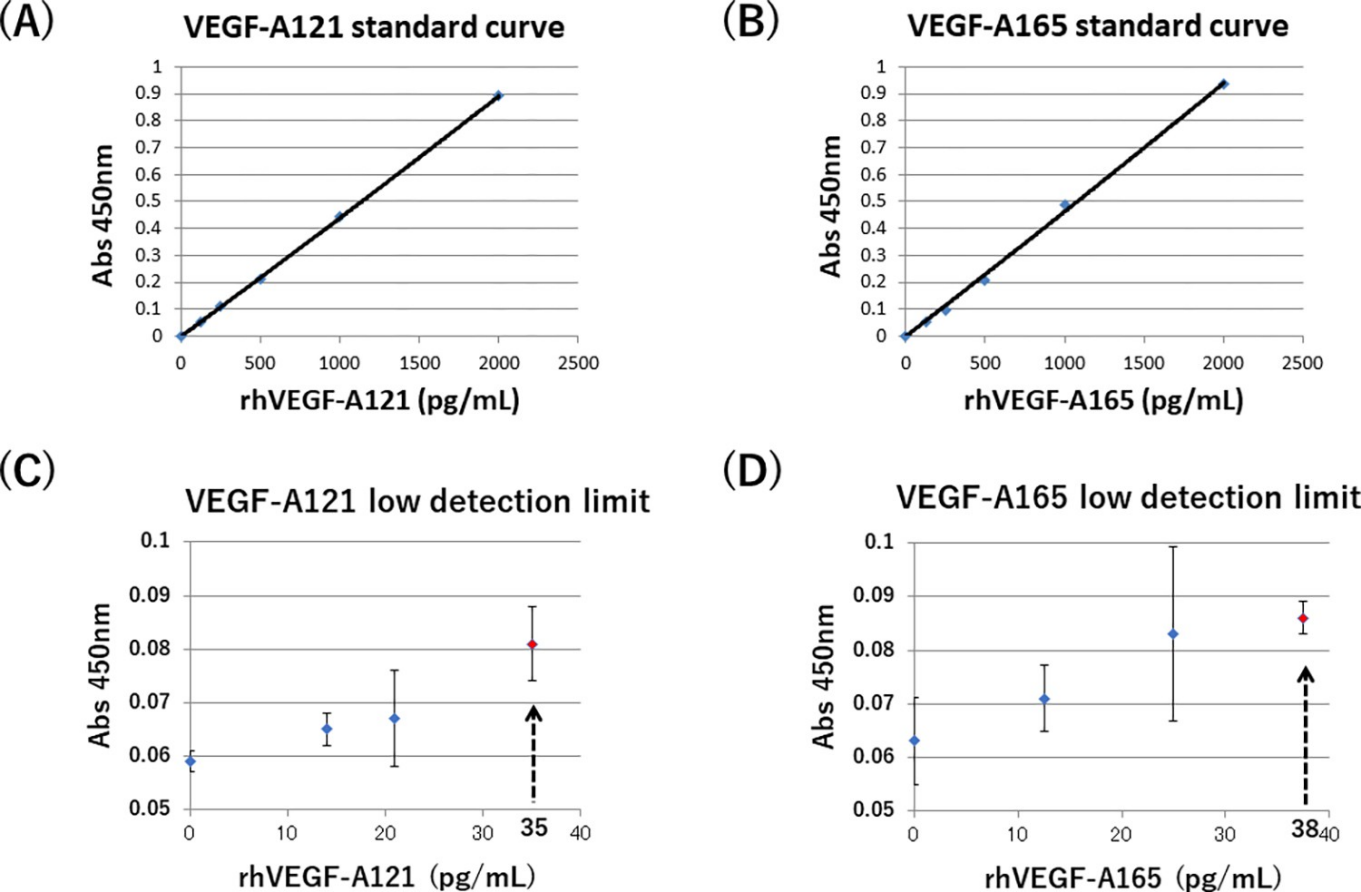
Standard curves for VEGF-A121 ELISA and VEGF-A165 ELISA and low detection limits of each ELISA. (A, B) A standard curve of rhVEGF-A121 by VEGF-A121 ELISA (A) and a standard curve of rhVEGF-A165 by VEGF-A165 ELISA (B) were independently created. (C, D) Different lower detection limits for rhVEGF-A121 and rhVEGF-A165 were determined in each ELISA (n = 5); the ELISA lower detection limits for VEGF-A121 or VEGF-A165 were 35 pg/mL (C) or 38 pg/mL (D), respectively. Abs 450 nm represents absorbance at 450 nm.

VEGF-A121 or VEGF-A165 ELISA was established by using isoform specific monoclonal antibodies. VEGF-A121 ELISA could provide the measurement of rhVEGF-A121 in a dose dependent manner, but not that of rhVEGF-A165 or rhVEGF-A189 (Fig 2A). In contrast, VEGF-A165 ELISA could show the dose-dependent manner of rhVEGF-A165, however, it did not cross-react with rhVEGF-A121 or rhVEGF-A189 (Fig 2B). Moreover, another VEGF-A variant protein, recombinant human VEGF-A206 could not be detected by both of VEGF-A121 and VEGF-A165 ELISA (Fig 2C and 2D). A standard curve obtained from different doses of rhVEGF-A121 and rhVEGF-A165 were shown in Fig 3. The working range of these assay was between 0 and 2,000 pg/mL (Fig 3A and 3B). The lower detection limit of this ELISA method was defined as the mean (± 2.6 S.D.) concentration of minimum rhVEGF-A121 and rhVEGF-A165. Accurate measurement of rhVEGF-A121 and rhVEGF-A165 could be achieved at concentration above 35 pg/mL (Fig 3C) and 38 pg/mL (Fig 3D), respectively.

### Measurement of hVEGF-A121 and hVEGF-A165 in cell condition media

**Fig 4:**
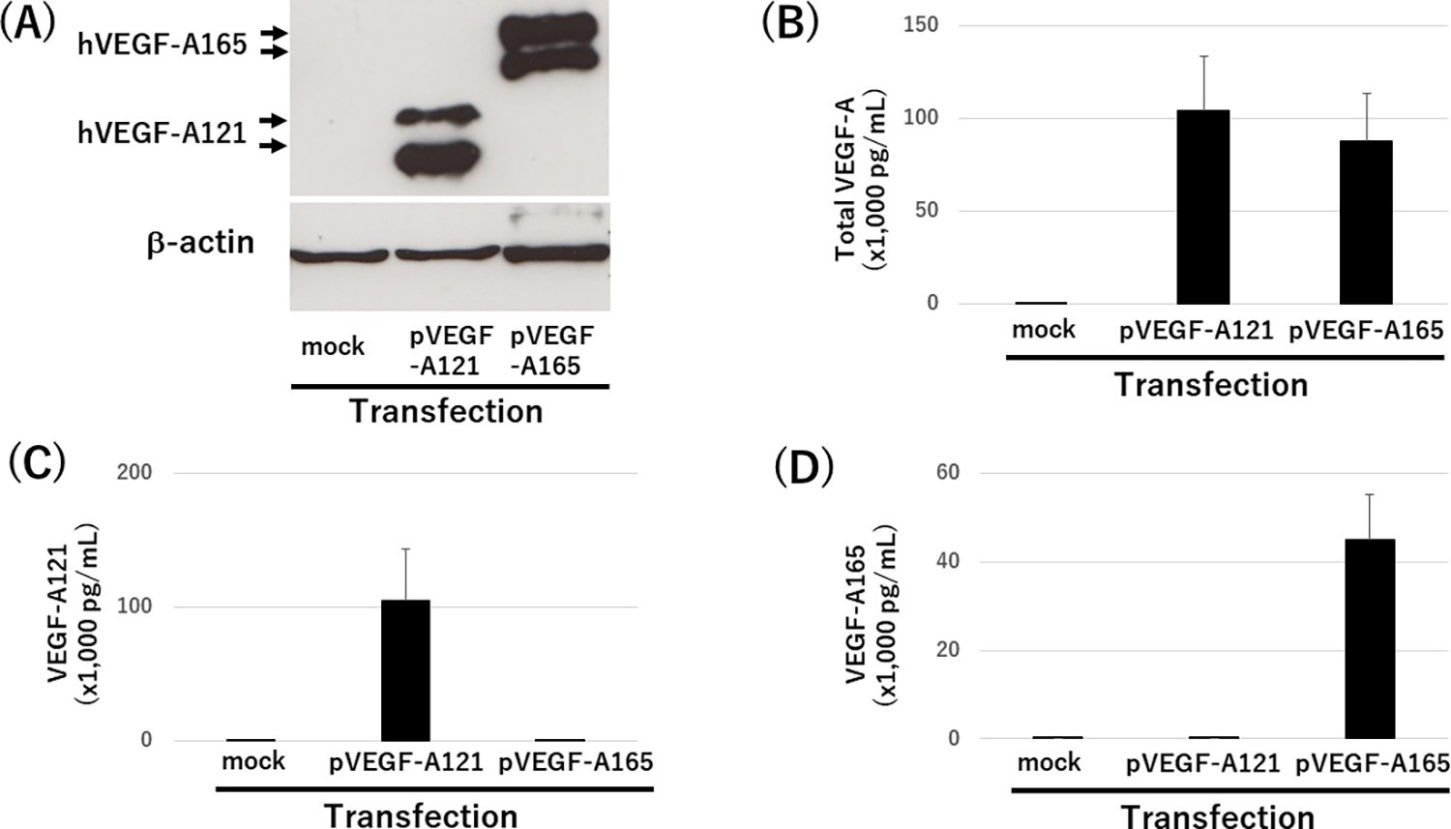
Detection of hVEGF-A121 and hVEGF-A165 in cell culture media by western blotting and isoform specific ELISA. (A) HEK293 cells were transfected with plasmids overexpressed VEGF-A121 (pVEGF-A121) and VEGF-A165 (pVEGF-A165) and empty plasmid (mock). Their cell lysates were blotted by VEGF-A polyclonal antibody and β-actin. (B—D) Total VEGF-A in the media from (A) was measured by Human VEGF Quantikine ELISA Kit (B), VEGF-A121 ELISA (C), and VEGF-A165 ELISA (D). Graph represents mean ± SD; n = 9.

HEK293 cells were transfected with hVEGF-A121 or hVEGF-A165 expression plasmid and cultured for two days. Their conditioned media were collected and blotted with VEGF-A polyclonal antibody. Conditioned media from hVEGF-A121 or hVEGF-A165 overexpressed cells proved to have only hVEGF-A121 and hVEGF-A165 recombinant protein, respectively (Fig 4A). When the same media described above were applied to the total VEGF-A, VEGF-A121, and VEGF-A165 ELISAs, no cross-reactivity was observed in the VEGF-A121 and VEGF-A165 ELISAs (Fig 4B–4D).

### Stability and precision of established ELISA for hVEGF-A121 or hVEGF-A165

Serum or plasma from healthy volunteers was frozen at -80 ˚C and thawed once, twice, and three times to evaluate the effects of multiple thawing. Repeated freeze-thaw cycles did not change the results for VEGF-A121 and VEGF-A165 values, with an average recovery of 94.5% (range = 97–92%) after two freeze-thaws and 100% (range = 112–88%) after three cycles. (S2A Table). The recovery of rhVEGF-A121 or rhVEGF-A165 added to pooled plasma and serum were 80–115% (S2B Table). To prove the precision of the value obtained from these ELISAs, plasma and serum samples with rhVEGF-A121 or rhVEGF-A165 concentrations of 100 and 500 pg/mL were analyzed for the assessment of intra-assay (n = 10) and inter-assay precision (n = 10).　The intra- and inter-assay coefficient of variations (CVs) for ELISAs of hVEGF-A121 and hVEGF-A165 were 3.1–8.1% and 4.8–9.1%, respectively (S2C Table).

### Measurement of VEGF-A121 and VEGF-A165 in serum and plasma of healthy volunteers

**Fig 5:**
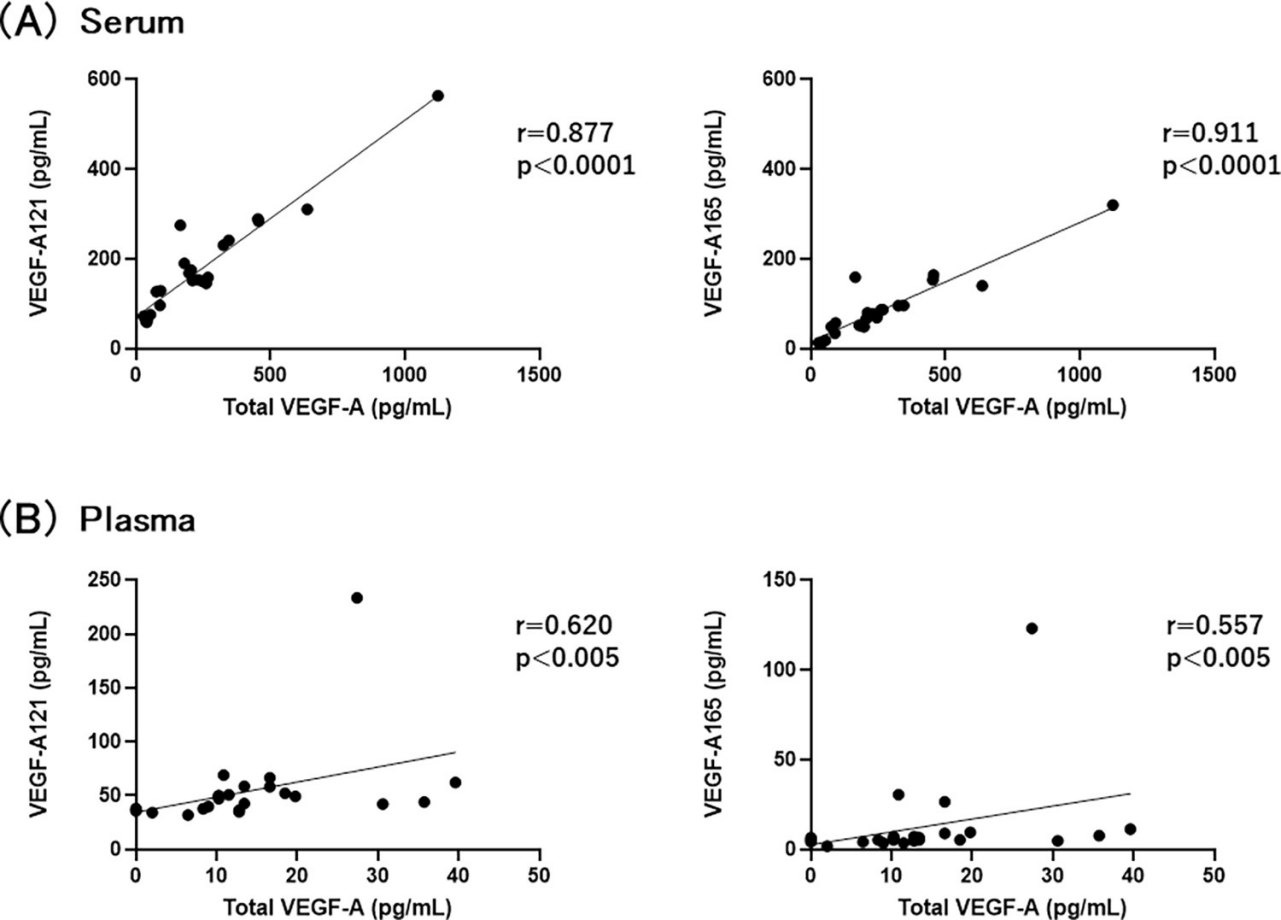
Levels of VEGF-A121 and VEGF-A165 were positively associated with total VEGF-A in human serum and plasma. Concentrations of total VEGF-A, VEGF-A121, and VEGF-A165 were measured in serum and plasma from healthy volunteers (n = 20). Correlation between total VEGF-A and VEGF-A121 or VEGF-A165 in serum (A) and plasma (B) were evaluated by a linear regression analysis (left panels of A and B: total VEGF-A vs. VEGF-A121, right panels of A and B: total VEGF-A vs. VEGF-A165). Correlations were determined using Spearman’s correlation test (two-tailed). Results are represented as significant at p < 0.05.

**Fig 6:**
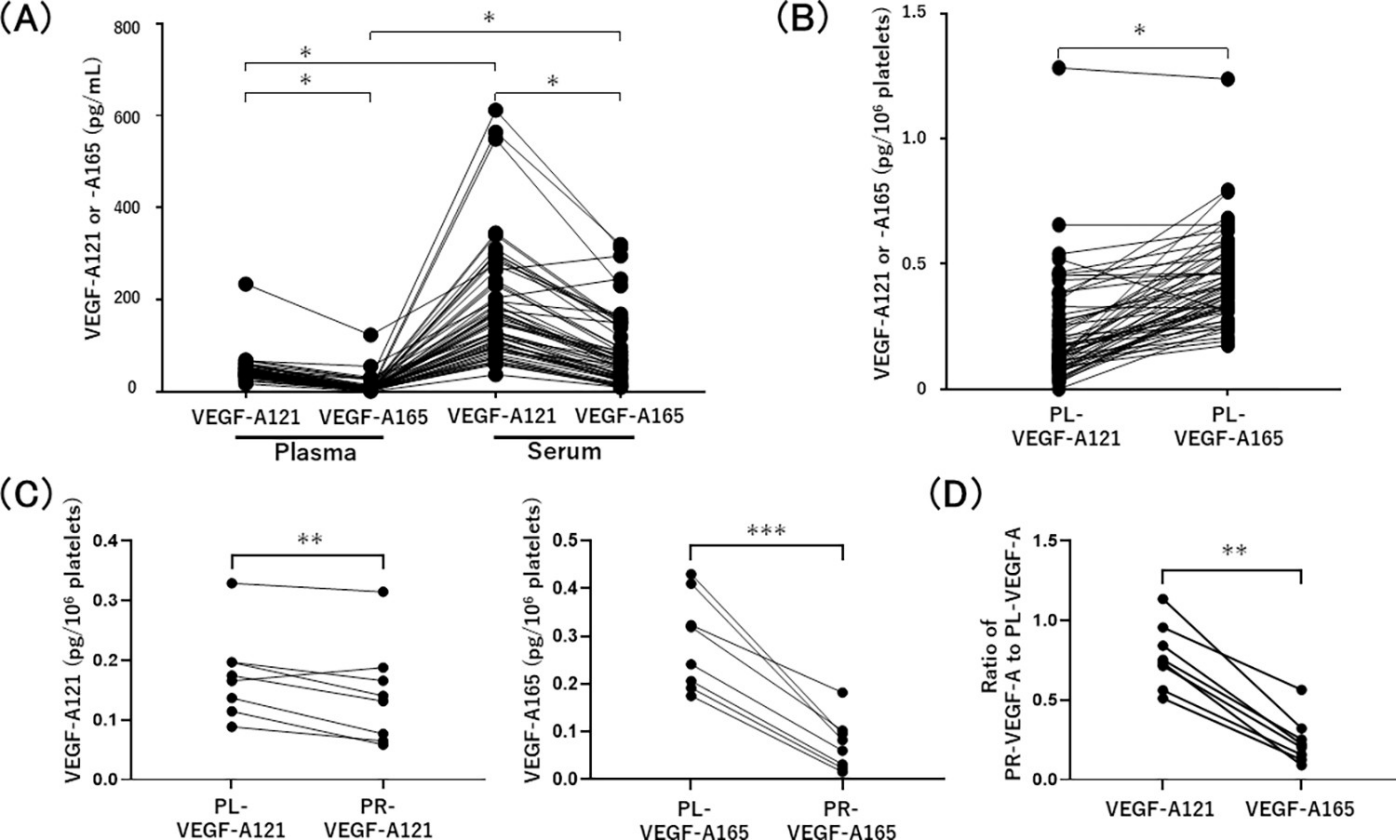
VEGF-A165 is more abundant than VEGF-A121 in human platelets. (A, B) VEGF-A121 and VEGF-A165 in plasma and serum (A) and those in platelet lysate (PL-VEGF-A121 and PL-VEGF-A165) (B) were measured by isoform specific ELISAs (n = 59). (C) VEGF-A121 released from aggregating platelets (PR-VEGF-A121) and VEGF-A121 in platelet lysate (PL-VEGF-A121) were the same levels for 10^6^ number of platelets (left panel). VEGF-A165 released from aggregating platelets (PR-VEGF-A165) was significantly lower than VEGF-A165 in platelet lysate (PL-VEGF-A165) (right panel) (n = 8). (D) The ratio of PR-VEGF-A121 to PL-VEGF-A121 and that of PR-VEGF-A165 to PL-VEGF-A165 in the same number of platelets were calculated from (C). *P < 0.0001, **P < 0.05, and ***P < 0.001.

Concentrations of VEGF-A121 and VEGF-A165 in plasma and serum from 59 healthy volunteers were measured by these established ELISAs. Total VEGF-A was measured by a commercial VEGF-A ELISA kit (Human VEGF Quantikine ELISA kit, R&D Biosystems). First, we compared to total VEGF-A and VEGF-A121 or VEGF-A165 in serum and plasma. The levels of VEGF-A121 and VEGF-A165 were positively and strongly correlated with total VEGF-A in serum (Fig 5A). In plasma, VEGF-A121 and VEGF-A165 also had well association with total VEGF-A (Fig 5B). Next, the levels of VEGF-A121 and VEGF-A165 in plasma and serum were compared. The concentrations of VEGF-A121 in both plasma and serum were higher than those of VEGF-A165 (Fig 6A). VEGF-A121 and VEGF-A165 levels in serum were higher than those in plasma, respectively (Fig 6A). These results indicate that the ratio of VEGF-A121 to VEGF-A165 in plasma and serum tend to be relatively similar.

### The ratio of VEGF-A121 and VEGF-A165 in platelets is different from that in serum and plasma

Importantly, VEGF-A165 was higher than VEGF-A121 in platelet lysate (PL), though VEGF-A165 was lower than VEGF-A121 in plasma and serum (Fig 6B). Several previous reports have showed that serum VEGF-A is primarily platelet-derived [20–22], however, these results suggest that VEGF-A165 in serum and plasma is not entirely platelet-derived. To prove the source of VEGF-A121 and VEGF-165 in serum, the amounts of VEGF-A121 and VEGF-A165 in the supernatant released from platelets were compared to those inside the same number of platelets. The concentration of each VEGF-A isoform in the supernatants of PPP and PRP stimulated by thrombin and CaCl_2_ (th/Ca^2+^), which is enough for platelet aggregation, was measured and the amount of VEGF-A released from platelets (PR-VEGF-A) was calculated according to the formula described in methods. The level of VEGF-A121 per 10^6^ platelets released by aggregation (PR-VEGF-A121) was about the same as the level of VEGF-A121 within the same number of platelets (PL-VEGF-A121) (Fig 6C). However, VEGF-A165 per 10^6^ platelets released by aggregation (PR-VEGF-A165) was significantly lower than VEGF-A165 within the same number of platelets (PL-VEGF-A165) (Fig 6C). Namely, the ratio of VEGF-A165 released from platelet aggregation to VEGF-165 within the same number of platelets is significantly lower than for VEGF-A121 (Fig 6D). These results suggest that the VEGF-A165 released by platelet aggregation is part of the platelet-intrinsic VEGF-A165, meaning that the amount of VEGF-A165 in serum does not fully reflect VEGF-A165 in platelets.

## Discussion

VEGF-A regulates angiogenesis through modulating endothelial cellular function, including proliferation, migration, and tubulogenesis [1]. Heterozygous VEGF-A knockout mice, which decreased expressions of all VEGF-A isoforms, exhibited embryonic lethal due to a dysregulation of vascular network [23, 24]. In contrast, VEGF-A transgenic mice showed a variety of phenotypes by their VEGF-A isoforms. Transgenic mice overexpressing VEGF-A specific in different tissues, including retina, keratinocytes, lymphocytes, and smooth muscle cells, has been reported [25–28]. These mice demonstrated unique phenotypes; increased new vessels originating from existing capillary [25], promoted inflammation [26], dysregulated immunomodulating system [27], and increased insulin-producing cells [28], depended on their specific tissues. These findings suggested that VEGF-A has a variety of ability to regulate vascular and non-vascular functions and raised an important question whether different VEGF-A isoforms control their own cellular functions.

There has been accumulating evidence that VEGF-A121, VEGF-A165, and VEGF-A189 contribute to many kinds of vascular activities separately. Each VEGF-A isoform produces tumors with vastly different vascularization patterns, vessel wall structure, and barrier function. Mouse VEGF-A188 (human VEGF-A189) overexpressing fibrosarcoma displayed completely different maturity of vasculature compared to fibrosarcoma overexpressing mouse VEGF-A120 (human VEGF-A121) or mouse VEGF-A164 (human VEGF-A165) in tumor transplantation mice models [29]. In human melanoma cell line, WM1341B, the angiogenesis types of VEGF-A121 and VEGF-A165 showed different; VEGF-A121 induced spare and peripheral tumor vascularization, however, VEGF-A165 exhibited dense and disorganized tumor vasculature [30]. Therefore, the ratio of VEGF-A121, VEGF-A165, and other VEGF-A isoforms must be clinically valuable for cancer progression and prognosis.

Tumor cells as well as blood cells, such as platelets, megakaryocytes, and monocytes, produce and release VEGF-A, which are detected in blood. A cancer therapy adding a humanized anti-VEGF-A monoclonal antibody, such as bevacizumab, to standard chemotherapy has been established in several cancers [31–33]. In gastric and pancreatic cancers, plasma VEGF-A would be a biomarker to predict clinical outcome in patients treated with bevacizumab [34, 35]. The association between mRNA expression of VEGF-A121 or VEGF-A165 and clinical outcome in cancer patients has been discussed [10–12], however, the protein levels of VEGF-A121 and VEGF-A165 in plasma or serum have not widely been demonstrated due to the lack of VEGF-A isoform specific measurement systems. Thereby, the evaluation of VEGF-A isoform in blood and clinical outcome could not be performed.

VEGF-A121 and VEGF-A165 are the major isoforms expressed in the most of human tissues and cancers [6]. Recently, measurement of VEGF-A121 in patients with certain cancers has been found to be useful for their severity and prognosis. In cancer tissue from patients with colorectal cancer, VEGF-A121 was more highly expressed than VEGF-A165, and tumors with high expression of VEGF-A121 showed increased tumor vascularity [36]. Plasma VEGF-A121 in patients with recurrent glioblastoma was higher than in healthy controls, and VEGF-A121 levels were associated with prognosis in these patients [37]. In addition, experiments using rat intracranial xenografts of human glioblastoma cells showed a positive correlation between VEGF-A121 and tumor size [37]. Therefore, independent measurement of VEGF-A121 and VEGF-A165 is important in the clinical evaluation of cancer patients.

Most of commercially available ELISAs to measure human VEGF-A concentrations detect both hVEGF-A121 and hVEGF-A165 (e.g., Human VEGF Quantikine ELISA Kit, R&D systems). Few ELISA kit that individually recognize these two VEGF-A specific isoforms has been commercially available (e.g., VEGF121 ELISA Assay, Cloud-Clone Corp. Houston, TX). Therefore, we created two monoclonal antibodies more specific to hVEGF-A121 or hVEGF-A165 and developed ELISAs by applying these antibodies. Our data showed no cross-reaction at least between hVEGF-A121 and hVEGF-A165 by the current ELISAs. To compare data from the current VEGF-A121 ELISA with those of commercially available ELISA kits for VEGF-A121, VEGF-A121 in serum from healthy controls was measured using the VEGF121 ELISA Assay kit (SEB851Hu, Cloud-Clone Corp). Although the VEGF-A121 measured by the commercial ELISA kit was considerably lower than the value obtained by the current VEGF-A121 ELISA, there was a strong correlation between the VEGF-A121 from these two ELISAs (S1 Fig). Further studies are needed to clarify the differences between commercial ELISA kits for VEGF-A121 and VEGF-A165 and the current ELISAs. However, we believe that the current ELISAs have a certain level of specificity and quantitation to measure VEGF-A121 and VEGF-A165 separately.

Isoform specific measurement of VEGF-A121 and VEGF-A165 revealed that the amount of VEGF-A121 was more than VEGF-A165 in serum and plasma of healthy volunteers, suggesting that VEGF-A121 might be the predominant splicing variants in adults without diseases (Fig 6A). Platelet is one of the major storage sources of VEGF-A and serum VEGF-A derives from the activated platelets [20–22]. Consequently, serum VEGF-A tends to be higher than plasma VEGF-A [38]. This previous finding is consistent with our data showing that VEGF-A121 and VEGF-A165 concentrations in serum are more plenty than those in plasma (Fig 6A).

Surprisingly, we found that platelets have greater amount of VEGF-A165 compared to VEGF-A121 (Fig 6B), although VEGF-A121 was higher than VEGF-A165 in serum as described above. To resolve this discrepancy, we collected supernatants of PRP and PPP after aggregating platelets completely by excess amount of thrombin and calculated platelet releasing VEGF-A121 (PR-VEGF-A121) and VEGF-A165 (PR-VEGF-A165). VEGF-A121 released from aggregating platelets (PR-VEGF-A121) and that in platelet lysate (PL-VEGF-A121) were almost the same levels for 10^6^ number of platelets, however, VEGF-A165 released from aggregating platelets (PR-VEGF-A165) was lower than that in platelet lysate (PL-VEGF-A165) (Fig 6C and 6D). One plausible explanation is that platelets might release all VEGF-A121 and some of VEGF-A165, which indicates the separate mechanisms of VEGF-A121 and VEGF-A165 secretions. Another hypothesis is that VEGF-A165 can be completely released together with VEGF-A121, and that some of secreted VEGF-A165 bind to platelet membranes or coagulation clots and cannot be measured in serum. Further studies will be necessary on this issue.

Traditionally, VEGF-A in serum and plasma has been the subject of research as a biomarker for cancers and other diseases. As our data showed, the ratio of VEGF-A121 to VEGF-A165 in platelets is different from that in serum and plasma, suggesting that this must be very important for clinical studies as a biomarker in the future.

## Conclusion

A new isoform specific ELISA system to detect hVEGF-A121 and hVEGF-A165 separately was produced by using isoform specific monoclonal antibodies. VEGF-A121 was abundant in serum and plasma, while VEGF-A165 was highly existed in platelets. This different distribution of VEGF-A121 and VEGF-165 between plasma/serum and platelets must be useful information considering as a biomarker for diseases in the future.

## Supporting information

S1 Fig Comparison of VEGF-A121 in serum among two different ELISAs. The figure shows the significant correlation of serum level of VEGF-121 measured by between the current ELISA and a commercial kit (SEB851Hu, Cloud-Clone Corp.). Linear regression, r^2^ = 0.7795, p = 0.0001 (n = 12).
(TIF)

S1 Table Monoclonal antibodies against human VEGF-A121 and VEGF-A165. (A) Amino acid sequences of human VEGF-A121 and VEGF-A165 antigen peptides. (B) Amino acid sequences of variable regions in human VEGF-A121 and VEGF-A165 monoclonal antibodies.
(TIF)

S2 Table Validation and Standardization of ELISA. (A, B) Stability of VEGF-A121 and VEGF-A165 ELISA by freeze and thaw cycles (A) and Spike and recovery test (B). (C) Precision of VEGF-A121 and VEGF-A165 ELISA measured by intra-assay and inter-assay. AVE; Average, SD; Standard Deviation, and CV; Coefficient of Variation.
(TIF)

S1 Raw images (PDF)
