# Supplementary figures and images for: VEGF-A165 is the predominant VEGF-A isoform in platelets, while VEGF-A121 is abundant in serum and plasma from healthy individuals
Source: PLoS One. 2023 Apr 7;18(4):e0284131. doi: 10.1371/journal.pone.0284131 (PMC10081782; doi:10.1371/journal.pone.0284131)

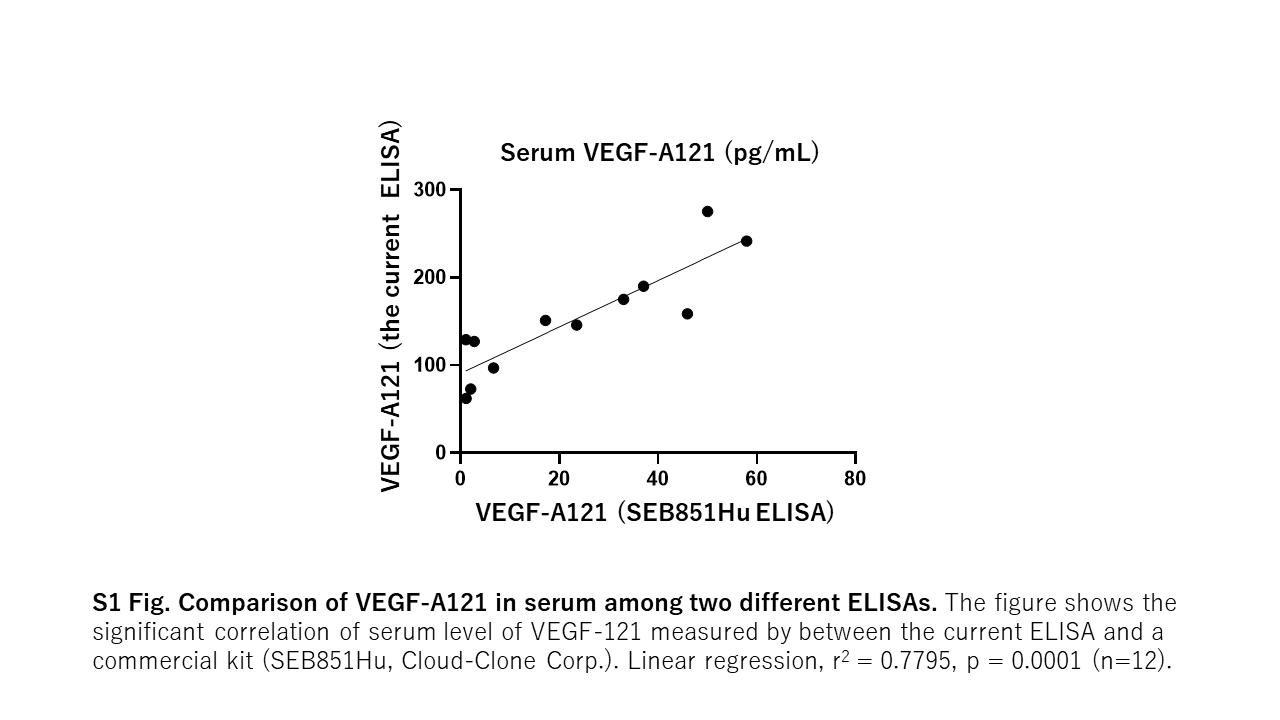

Supplement: S1 Fig — The figure shows the significant correlation of serum level of VEGF-121 measured by between the current ELISA and a commercial kit (SEB851Hu, Cloud-Clone Corp.). Linear regression, r2 = 0.7795, p = 0.0001 (n = 12). (TIF) [file pone.0284131.s001.tif]

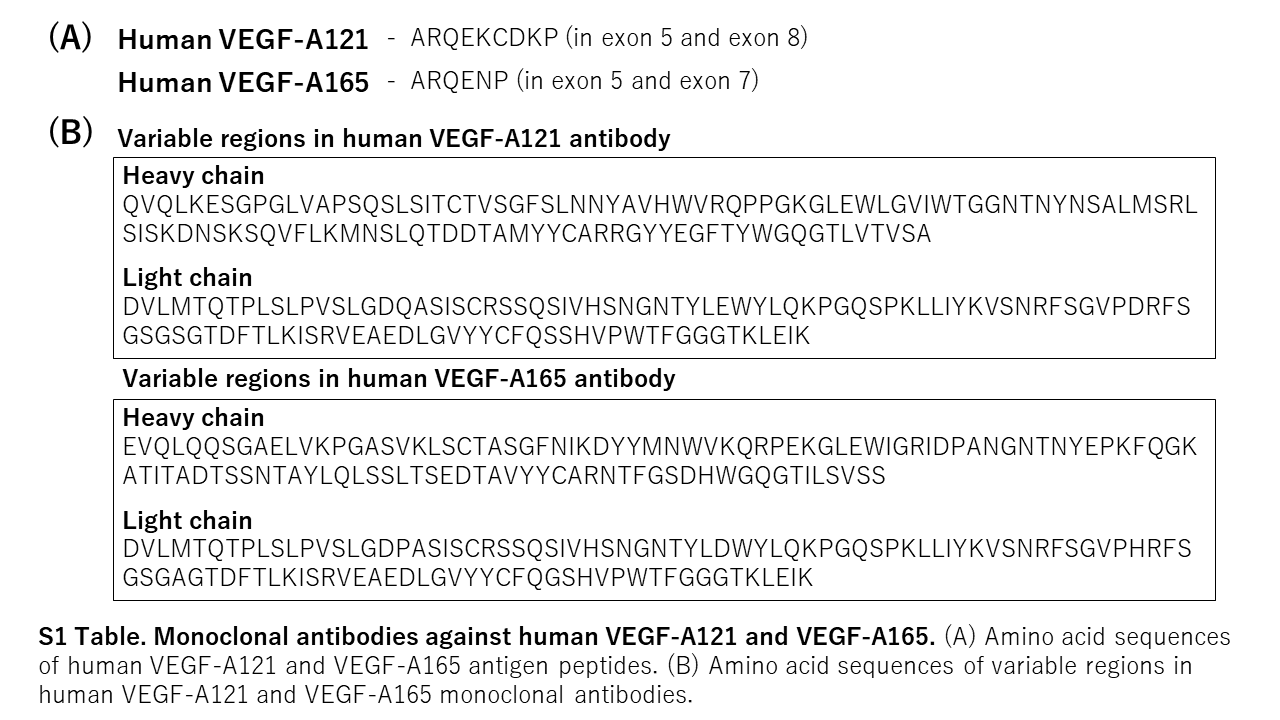

Supplement: S1 Table — (A) Amino acid sequences of human VEGF-A121 and VEGF-A165 antigen peptides. (B) Amino acid sequences of variable regions in human VEGF-A121 and VEGF-A165 monoclonal antibodies. (TIF) [file pone.0284131.s002.tif]

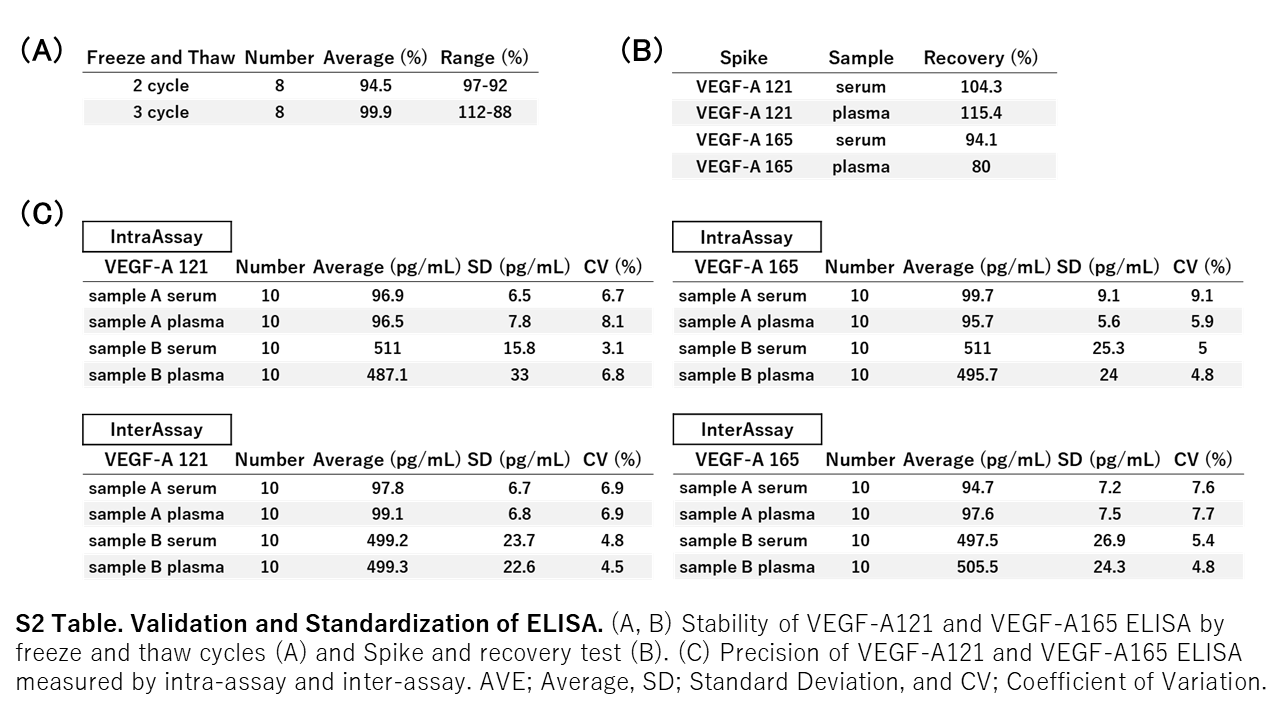

Supplement: S2 Table — (A, B) Stability of VEGF-A121 and VEGF-A165 ELISA by freeze and thaw cycles (A) and Spike and recovery test (B). (C) Precision of VEGF-A121 and VEGF-A165 ELISA measured by intra-assay and inter-assay. AVE; Average, SD; Standard Deviation, and CV; Coefficient of Variation. (TIF) [file pone.0284131.s003.tif]

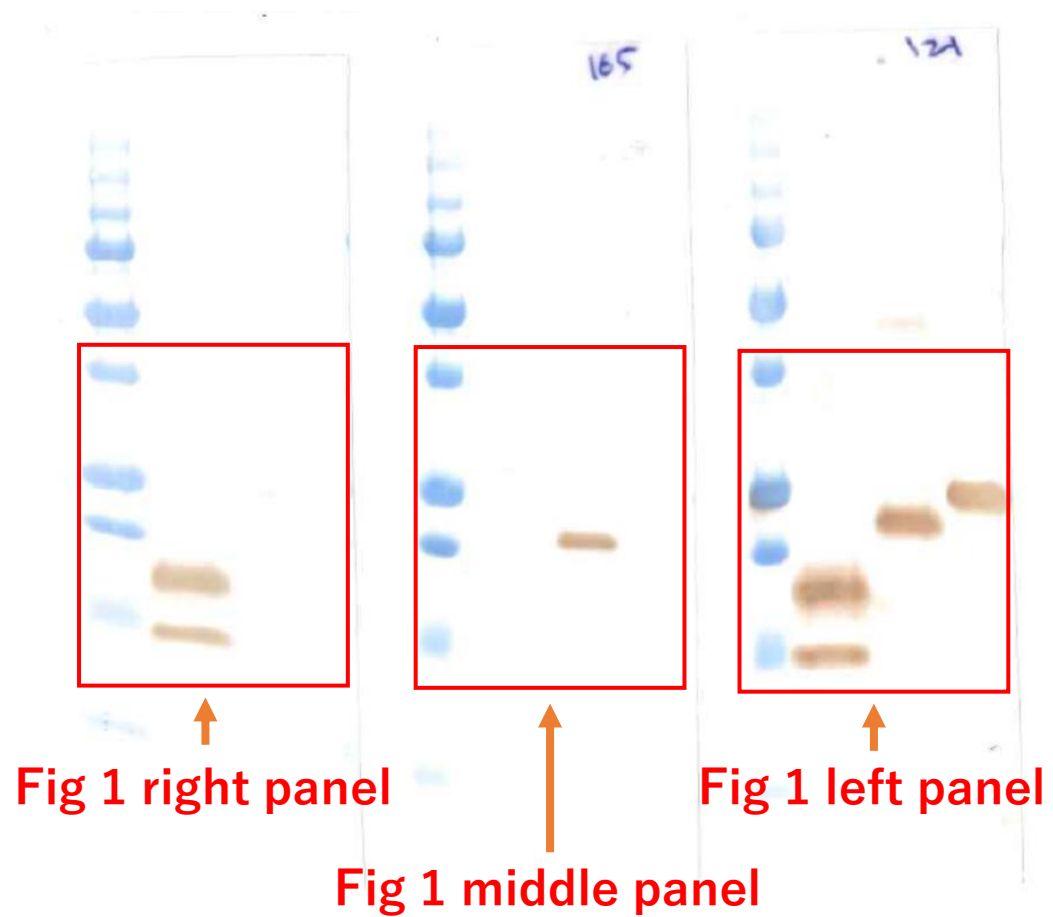

Original Blot for Figure 1

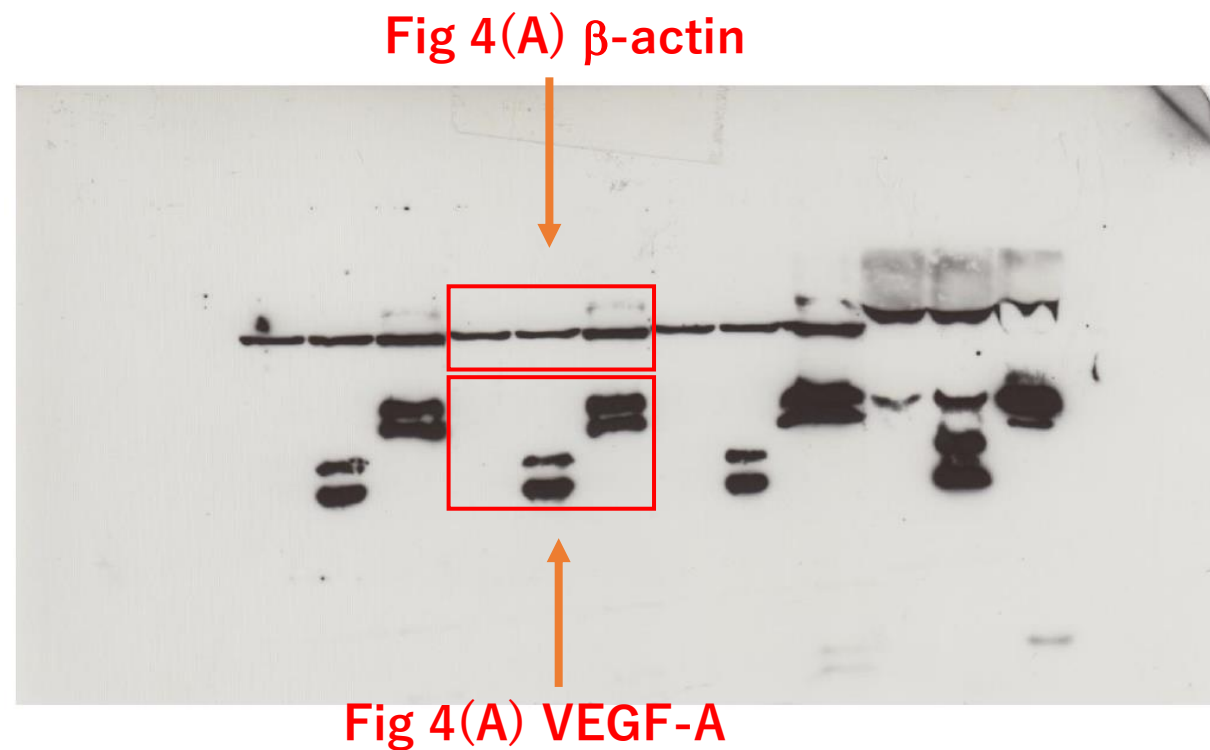

Original Blot for Figure 4(A)

Supplement: S1 Raw images — (PDF) [file pone.0284131.s004.pdf]
